# Natural variation and improved genome annotation of the emerging biofuel crop field pennycress (*Thlaspi arvense*)

**DOI:** 10.1093/g3journal/jkac084

**Published:** 2022-04-13

**Authors:** Tatiana García Navarrete, Cintia Arias, Eric Mukundi, Ana Paula Alonso, Erich Grotewold

**Affiliations:** 1 Department of Biochemistry and Molecular Biology, Michigan State University, East Lansing, MI, 48824, USA; 2 BioDiscovery Institute and Department of Biological Sciences, University of North Texas, Denton, TX, 76203, USA

**Keywords:** *Thlaspi arvense* (pennycress), genome annotation, accessions, RNA-seq, SNPs

## Abstract

The Brassicaceae family comprises more than 3,700 species with a diversity of phenotypic characteristics, including seed oil content and composition. Recently, the global interest in *Thlaspi arvense L.* (pennycress) has grown as the seed oil composition makes it a suitable source for biodiesel and aviation fuel production. However, many wild traits of this species need to be domesticated to make pennycress ideal for cultivation. Molecular breeding and engineering efforts require the availability of an accurate genome sequence of the species. Here, we describe pennycress genome annotation improvements, using a combination of long- and short-read transcriptome data obtained from RNA derived from embryos of 22 accessions, in addition to public genome and gene expression information. Our analysis identified 27,213 protein-coding genes, as well as on average 6,188 biallelic SNPs. In addition, we used the identified SNPs to evaluate the population structure of our accessions. The data from this analysis support that the accession Ames 32872, originally from Armenia, is highly divergent from the other accessions, while the accessions originating from Canada and the United States cluster together. When we evaluated the likely signatures of natural selection from alternative SNPs, we found 7 candidate genes under likely recent positive selection. These genes are enriched with functions related to amino acid metabolism and lipid biosynthesis and highlight possible future targets for crop improvement efforts in pennycress.

## Introduction

A case has been made for a second green revolution that involves the domestication of new crops with enhanced nutritional and/or industrial value, lower water and fertilizer inputs, and that do not compete with current staple crops (Wollenweber *et al.*, 2005; Mayes *et al.*, 2012; Østerberg *et al.*, 2017). While classical domestication and breeding have largely been responsible for the development of the approximately 200 plant species that are currently economically important, it can take decades to millennia to transform wild progenitor species into productive crops (Doebley *et al.*, 2006). In contrast, developments in genomics and genome editing are permitting to significantly accelerate this process through molecular and precision breeding (Moose and Mumm, 2008; Khan *et al.*, 2019). To enable these to take place, high-density markers, the availability of a genome sequence, and the possibility to edit the genome need to be in place.

The diploid winter annual *Thlaspi arvense* (field pennycress, pennycress from here on), a member of the Brassicaceae family and a close relative of *Arabidopsis*, has been shown to accumulate seed oils that make it ideally suited for biodiesel and jet fuel production (Moser *et al.*, 2009; Al-Shehbaz, 2012; Fan *et al.*, 2013; Sedbrook *et al.*, 2014). Pennycress is being developed as a new winter annual cash crop cover with a short enough seed-to-seed cycle that permits it to take advantage of unused farmland in between maize or soybean crops (Sedbrook *et al.*, 2014; McGinn *et al.*, 2019). The pennycress genome is about 539 Mb, 4 times larger than that of *Arabidopsis* (∼135 Mb), and is organized into 7 chromosomes (2*n *=* *14). Transcriptomes and a draft genome from the winter annual line MN106 were generated (Dorn *et al.*, 2013, 2015) and recently complemented by whole genome sequencing of several accessions (Dorn *et al.*, 2018), as well as sequencing of inbred line Spring 32-10 (McGinn *et al.*, 2019). Besides a draft genome being available, pennycress can be easily transformed using *Agrobacterium*-mediated floral dip (McGinn *et al.*, 2019), at efficiencies comparable to those of *Arabidopsis* (Clough and Bent, 1998). Forward and reverse genetic tools are becoming rapidly available and will provide pennycress all the advantages that have made *Arabidopsis* such a useful reference plant (Chopra *et al.*, 2018).

Here, we describe an integrative strategy for improving the pennycress genome annotation using genome-guided and *de novo* transcriptome assembly with the aim to identify possible new transcripts, confirm gene models and identify potential isoforms. For this, we used the combination of public data and data produced for 22 pennycress accessions derived from embryos at 2 developmental stages and combined with long-reads obtained by the Oxford Nanopore Technology. Transcriptomes from pennycress embryos are an excellent resource for understanding expression patterns at a critical developmental stage, where the plant carries on essential processes such as oil biosynthesis and lipid storage. Therefore, we also identified single-nucleotide polymorphisms (SNPs) from RNA-seq data to identify possible natural variations with potential use in pennycress breeding programs. In addition, we used identified SNPs to evaluate likely signatures of natural selection and identify 7 genes under possible positive selection with functions related to amino acid and lipid metabolism. The addition of transcriptome information from several accessions in a tissue that is important from a biotechnological perspective provides an important contribution to the current knowledge base on this emergent oilseed crop.

## Materials and methods

### Plant growth, RNA extraction, and RNA-seq analysis

Twenty-two natural variants of *T.* *arvense* sourced from distinct geographic regions were selected from the National Germplasm System (Table 1). The different lines were germinated in plates containing Murashige and Skoog medium supplemented with 1 mM G4/G7 gibberellins and using Whatman paper as support. The germinated seeds were transferred to pots and grown in a growth chamber at 22°C, with 200 µmol/m^2^/s light intensity and a 16 h/8h day/night cycle until the emergence of the first true leaves. All the lines were considered winter varieties, therefore the pots were transferred to a cold room (4 °C) for 3 weeks under low light conditions (100 µmol/m^2^/s light intensity and 10 h/14 h day/night cycle) to induce flowering. Finally, the different lines were grown in a greenhouse where the temperature was controlled between 21 and 25°C, the photoperiod was 16 h/8h and the light intensity was kept close to 300 µmol/m^2^/s.

**Table 1.** List of pennycress accession used in this study.

Reference name	Accession name	Origin	Illumina high-quality read counts		Nanopore read counts	Alignment rates (%)		
			10 DAP	16 DAP	10 DAP	Illumina		Nanopore
						10 DAP	16 DAP	10 DAP
PC1	Ames 32908	Illinois, USA	166,159,412	142,642,262		98.3	98.4	
PC2	Ames 32872	Armenia	147,309,695	147,941,054		96.3	96.1	
PC3	Ames 31499	British Columbia, Canada	158,493,892	148,009,513		98.3	98.5	
PC4	Ames 31497	Saskatchewan, Canada	161,944,688	147,716,867		98.3	98.5	
PC5	Ames 29512	Ohio, USA	145,456,696	148,280,855		98.2	98.3	
PC6	Ames 31026	Colorado, USA	158,043,152	150,666,428		98.1	98.2	
PC7	Ames 31501	Manitoba, Canada	157,497,428	152,667,158		98.4	98.4	
PC8	Ames 31500	Alberta, Canada	161,851,429	131,646,039		98.4	98.2	
PC9	Ames 31488	Ontario, Canada	139,200,591	149,532,332		98.3	98.2	
PC10	Ames 30933	Magallanes, Chile	128,441,416	134,602,498		97.6	97.5	
PC11	Ames 30985	South Dakota, USA	127,281,910	131,307,530		97.6	97.5	
PC12	Ames 24499	Former Serbia and Montenegro	128,470,022	135,033,559		97.6	97.5	
PC13	Ames 29531	North Dakota, USA	115,343,123	151,205,534		98.1	98.4	
PC14	Ames 22461	Poland	128,876,158	99,471,991		97.7	97.3	
PC15	PI 650287	Bas-Rhin, France	131,295,933	132,096,941		97.6	97.0	
PC16	PI 633415	Saxony, Germany	131,262,050	132,955,028		97.6	96.5	
PC17	PI 650284	Thuringia, Germany		131,349,471			97.4	
PC18	Ames 30982	Iowa, USA	147,434,032	278,054,333		97.6	97.4	
PC19	Ames 31012	Colorado, USA	131,144,003	133,595,159		97.5	96.	
PC20	Ames 31498	Alberta, Canada	128,261,227	133,677,160		98.0	96.2	
PC21	PI 650285	Saxony, Germany	150,084,778	151,364,594		98.2	98.2	
PC22	MN106	Minnesota, USA	147,772,792	148,588,444	10,272,041	98.0	98.0	84.7

The pennycress flowers were hand-pollinated and new flowers were tagged every day to stage embryo development. Two embryo stages that correspond to the beginning [10 days after pollination (DAP)] and middle (16 DAP) points of the pennycress seed oil accumulation curve (Tsogtbaatar *et al.*, 2015) were selected for the study. For that, the immature pods were collected at 10 and 16 DAP, the seeds dissected by removing seed coats and endosperm, and the embryos were collected in liquid nitrogen. At least 3 biological replicates were used for each development stage for each pennycress accession. The embryos were ground frozen and RNA was extracted using a buffer containing 2% CTAB, 2 mol/l NaCl, 100 mM Tris-HCL (pH = 8), 3% beta-mercaptoethanol, 25 mM EDTA, 0.5 g/l spermindine, and 3% polyvinylpolypyrrolidone at 65°C for 10 min. To clean the RNA, 2 extractions with chloroform:isoamyl-alcohol (24:1, v/v) were performed. Then, a first precipitation step was done by adding 40 µl of sodium acetate 3.2 M (pH 5.5), 800 µl of ethanol 96%, and 50 µg of glycogen. After overnight incubation at −20°C, followed by 1 h of centrifugation at 13,000 × *g* and 4°C, the pellet was resuspended in RNAse-free water. For a second precipitation step, lithium chloride was added to 2.5 M and incubated overnight at −20°C. The samples were then centrifuged at 13,000 × *g* and 4 °C for 1 h, and the pellets were resuspended in RNAse-free water. Subsequently, DNA contamination was eliminated by treating the RNA with Qiagen RNase-Free DNase and followed by a concentration step with RNeasy MinElute Cleanup Kit (Qiagen).

The quality and concentration of total RNA were evaluated with Qubit 2.0 (Life Technologies) and by capillary electrophoresis using a bioanalyzer (Agilent). RNA-seq libraries were sequenced with Illumina HiSeq 2500 (Illumina, USA) as a paired-end with a length of 150 bp. For each developmental stage and pennycress accession, 3 or more biological replicates were obtained, with the exception of accession PI 650284 for which only the 16 DAP stage was analyzed. Illumina reads were evaluated with FastQC v.0.11.9 (Andrews, 2010) and trimmed for quality with Trimmomatic v.0.39 (Bolger *et al.*, 2014). Then, the clean reads were used to generate genome-guided and *de novo* transcriptome assembly for each sample. For the genome-guided transcriptome assembly, the reads were aligned with default parameters to the reference pennycress genome (Dorn *et al.*, 2015) with HISAT v2.0.4 (Kim *et al.*, 2015) and each alignment file in bam format was used as input in StringTie v.2.1.1 (Pertea *et al.*, 2015). In addition, we incorporated previously-generated RNA-seq data (Dorn *et al.*, 2013), into a set of transcripts.

For *de novo* assembly, the transcriptome from each line was assembled with Trinity v2.8.4 (Grabherr *et al.*, 2011) using *in silico* normalization and default parameters. Each *de novo* assembly was used as input in BUSCO v.3.1.0 (Simão *et al.*, 2015) for evaluating the gene content of single-copy orthologs using the Embryophyta library OrthoDB v8 database (Kriventseva *et al.*, 2015). The genome-guided transcriptome assembly and *de novo* transcriptome were incorporated in PASA v.2.4.1 (Haas *et al.*, 2003) to build a comprehensive transcriptome database. Next, in the first step of PASA, the transcripts were aligned with GMAP (Wu and Watanabe, 2005) to the reference pennycress genome (with the following parameters: g–min_per_ID 95, –min_per_aligned 80). Then PASA performed an annotation comparison between the previous annotation and the new annotation. Finally, PASA identified cases to do gene model updates. The annotation file generated by PASA was evaluated with Trinotate (Bryant *et al.*, 2017) and the statistics were obtained with NGSEP v.4.0.0 (Tello *et al.*, 2019) with the module TranscriptomeAnalyze.

### Long-read RNA sequencing and analysis

To improve the fidelity of the sequence obtained by RNA-seq, we subjected 1 sample of 10 DAP embryo total mRNA to Nanopore library preparation and sequencing. Libraries were prepared using the Nanopore PCS109—cDNA-PCR Sequencing Kit. The library was loaded onto an Oxford Nanopore FLO-MIN106D (vR9.4.1) flow cell and sequencing was performed using the GridION x5 instrument by the Research Technology Support Facility Genomics Core at Michigan State University. GridION software release 19.12.2 was used for data analysis. Real-time base calling of reads was performed by guppy_bascaller v3.2.8.

The quality control for Nanopore sequencing data was performed using MinIONQC v1.4.1 (Supplementary Table S1). Reads with a mean quality of ≥7 were subjected to an error correction phase with LoRDEC v.0.9 (Salmela and Rivals, 2014) together with the Illumina reads of the MN106 accession (parameters -k 19, -s 3). The Fastq file with the corrected reads generated by LoRDEC v.0.9 was aligned to the reference pennycress genome (v.1.0) (Dorn *et al.*, 2015) with minimap v.2 (Li, 2018). The alignment file in bam format was used as input in StringTie v.2.1.1 (Pertea *et al.*, 2015) (parameters -L -f 0.2), generating potential transcripts, which were reported in general feature format (gff3). These transcripts were evaluated with Transdecoder (Haas *et al.*, 2003). The transcripts with the completed open reading frame (ORF) were integrated with the annotation from RNA-seq data using the GFF3CombineAnnotations module of NGSEP v.4.0.0 (Tello *et al.*, 2019). The new annotation in gff3 format was used as input on StringTie v.2.1.1 (parameter-G) (Pertea *et al.*, 2015) and the bam files to obtain the measure of expression on transcripts per million TPM of each gene.

The functional annotation was carried out with Trinotate pipeline v3.1.1 (Bryant *et al.*, 2017), where all protein sequences were compared with known sequences from the Uniprot database using BLAST+ v2.9.0 (Camacho *et al.*, 2009). Furthermore, Trinotate detected the protein domain in the sequences evaluated with the Pfam database through HMMER v.3.1b2 (Eddy, 2011). Finally, the gene ontology's (GO) Trinotate report was used to compare with the GO annotations from *Arabidopsis thaliana* (The Arabidopsis Information Resource, 2021) using WEGO 2.0 (Ye *et al.*, 2018), where we obtained a summary file with the number of genes in each GO term.

### Pennycress genome analysis

For pennycress genome annotation, we used General Feature Format (gff3) files as input in the module TranscriptomeAnalyzer from NGSEP v.4.0.0 (Tello *et al.*, 2019). It generated statistics on the assembled transcriptome such as gene length, transcript length, number of exons, transcripts per gene, coding sequence (CDS) length, and protein length. To evaluate the pennycress annotation, we used the module TranscriptomeFilter from NGSEP v.4.0.0 (Tello *et al.*, 2019) to identify possible genes without the presence of start and stop codons.

### SNP identification from RNA-seq data and evaluation of the genes under selection

Variant calling analyses were carried out for each bam file with NGSEP v.4.0.0 (Tello *et al.*, 2019) with the parameter -maxBaseQS 30 -maxAlnsPerStartPos. Only SNPs with quality (GQ field) of 40 or more were retained. Initially, 60,607 SNPs were identified, of which approximately 57% were only identified in PC2. Then, we removed the PC2 private SNPs to have more data points with meaningful information in relation to the other accessions evaluated. The final VCF file was filtered according to the following parameters: minimum genotyping quality score (-q40), the minimum number of samples genotyped to keep the variant (-m22), and keep only biallelic SNVs (-S), the minimum distance between variant (-d 10). The variant call file (vcf) was used as input in the R library adegenet package (Jombart, 2008) to evaluate the genetic structure in 3 steps. First, a phylogenetic tree was generated through the neighbor-joining algorithm. One thousand bootstraps tested the branching support. Second, principal component analysis (PCA) was carried out to evaluate the divergence between the different pennycress accessions. Finally, we performed a multivariate statistical approach through discriminant analysis of principal components (DAPC) to evaluate each pennycress sample's posterior assignments. The individual assignment test was carried out with STRUCTURE v.2.3.4 (Pritchard *et al.*, 2000) using the “Admixture” model and correlated allele frequencies.

The gene selection analysis, along with the pennycress accessions analyzed, was estimated by clustering the samples according to genetic structure with the module VCFAlleleSharingStats of the NGSEP v.4.0.0 (Tello *et al.*, 2019). The results were filtered according to fixation indices (Fst), withholding genes with Fst ≥ 0.8. Genes under selection were identified by the Tajima's *D* test using the common measure of significance for Tajima's *D* below −2 (Korneliussen *et al.*, 2013). Genes related to fatty acids biosynthesis were obtained from aralip (Li-Beisson *et al.*, 2013) and analyzed based on a list of candidate genes related to synthesis and lipid storage.

## Results and discussion

### Structural and functional annotation improvement of the pennycress genome

In the current annotation of the pennycress genome (version 1, v1.0 from here on), 27,390 protein-coding genes were reported with an average length of 2,195 bp (Dorn *et al.*, 2015). An initial evaluation of this data showed that 10.7% (2,926) of the annotated protein-coding genes lack complete transcripts (with standard translation start and stop codons). Examples are provided by the putative genes *Ta1.0_00777* and *Ta1.0_04100* annotated as encoding transcription factors belonging to the TCP (Teosinte branched1/Cincinnata/Proliferating cell factor) and GRAS (GAI, RGA, SCR) families, respectively. These genes lack ATG translation start codons in the current version (Supplementary Fig. S1), highlighting the need to significantly improve the pennycress structural annotation. Also, we assessed this annotation through completeness with single-copy orthologs with the BUSCO program v.3.1.0 (Simão *et al.*, 2015). We found 1,362 genes; of them, 92.1% (1,327) are complete and single-copy, 2.43% (35) are fragmented genes, and 3.4% (49) are missing genes.

**Fig. 1.**
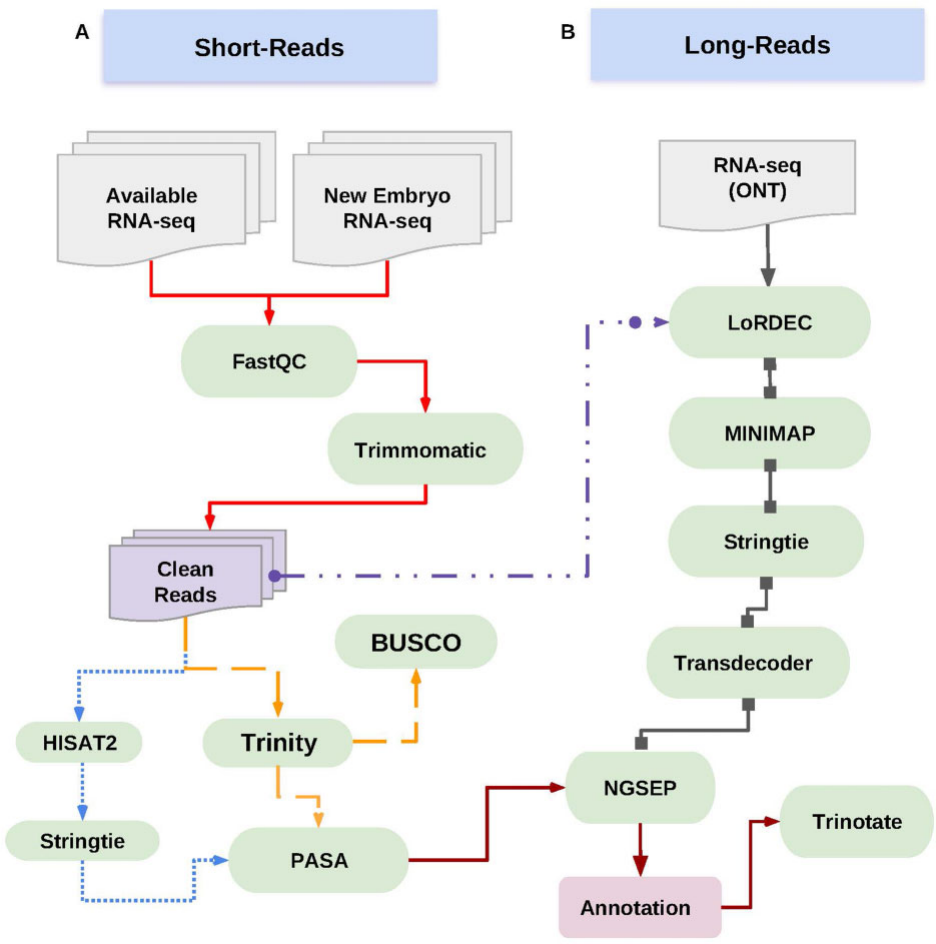
Workflow for the re-annotation of the pennycress genome. Regular oval shapes indicate the tools used for data processing, and the multi-document figure symbolize the input data from the Illumina and ONT sequencing platforms. (a) The dotted line represents the workflow for the genome-guided transcriptome assembly, and the double dashed line the de novo transcriptome assembly. (b) The line with a square arrow indicates the workflow incorporating the long reads, and the double dash dot line with circle arrow corresponds to the integration of short reads for the correction phase of the long reads.

To enhance the annotation of the pennycress genome, we performed extensive RNA-seq analyses on RNA obtained from embryos corresponding to 22 different accessions in biological triplicate at 2 developmental stages (10 and 16 DAP) for a total of 5,614,051,998 paired-end 100-bp reads (Table 1). One of the RNA extractions obtained from accession MN106 at 10 DAP was also subjected to Oxford Nanopore Technology (ONT) sequencing, resulting in a total of 10,272,041 reads (Table 1 and Supplementary Table S2). Our sequencing results were combined with publicly available RNA-seq (Supplementary Table S3) information (Fig. 1a), resulting in genome-guided and de novo transcriptome assemblies for each sample.

To assess the integrity of the *de novo* transcriptome assemblies, we used the BUSCO tool v.3.1.0 (Simão *et al.*, 2015). The percentage of the complete orthologs in each *de novo* transcriptome was around 85% (Supplementary Fig. S2), giving us confidence that our transcriptome analysis provides valuable information for annotation of the genome. Subsequently, we used Program to Assemble Spliced Alignments-PASA (Haas *et al.*, 2003) to build a comprehensive transcriptome database, with an assembly of different gene models and the corresponding isoforms. In addition, all the short reads obtained from RNA-seq results were used to correct possible sequencing errors in the long reads, by using the LoRDEC v.0.9 (Salmela and Rivals, 2014) tool. The error rate in the original long-reads was around 5%, and after the correction process, the error is reduced to 1% (Supplementary Fig. S3). The corrected long reads were aligned to the reference pennycress genome and used to generate potential transcripts. Finally, we integrated the predicted transcripts from long and short reads and generated the functional annotation of the transcripts (Fig. 1).

**Fig. 2.**
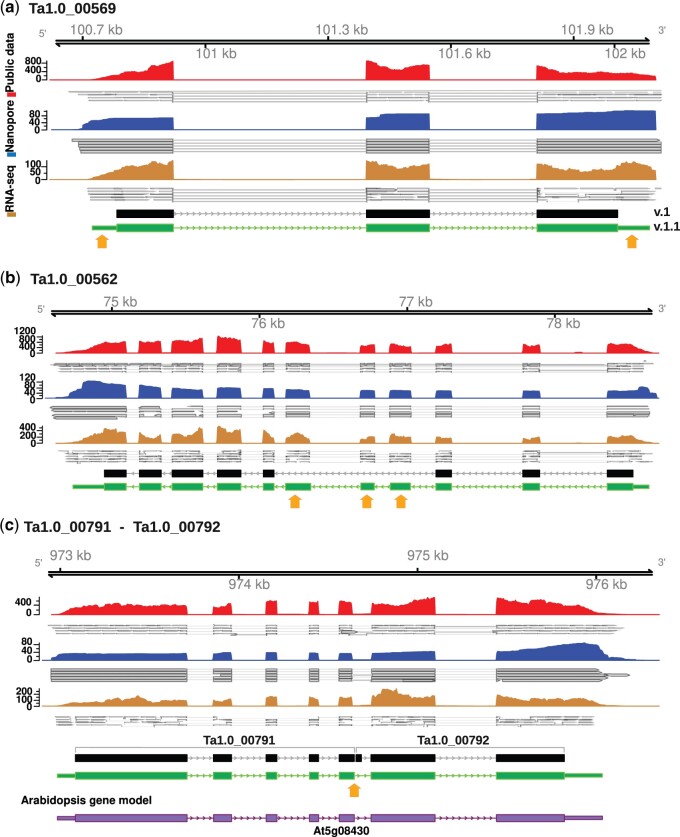
Examples of improved genome annotation. Genome viewer images indicating the reads obtained from the two sequencing platforms comparing gene models from the current genome (v.1.0) and as provided in this study (v.1.1). The first track shows read distributions corresponding to previously available RNA-seq data, the second track reads obtained from Nanopore, and the last track shows the reads obtained from Illumina embryo RNA-seq. (a) Example of a gene model in which the new sequencing data resulted in the addition of 5’UTR and 3’UTR regions. (b) Example of a gene model in which the new sequencing data resulted in identifying three additional exons. (c) Example of two adjacent gene models in which the new sequencing data showed that they actually corresponded to just one gene.

We identified a total of 27,213 protein-coding genes with an average length of 2,454 bps and an average protein length of 416 amino acids. Interestingly, of those sets of genes, 18,222 genes were supported by long-reads (Supplementary Table S6) and 459 corresponded to full-length transcripts (Supplementary Table S8). When we compared our results (version 1.1, v1.1 from here on) with the annotation provided by the pennycress genome (v1.0) (Table 2), we determined a concordance in 18,203 gene models. However, our analysis improved the gene models in various ways, specifically by adding 5′ and 3′ untranslated regions (UTRs) for 10,426 genes, significantly increasing the number of genes with annotated UTRs from the previous version in which only 5% of the genes (1,466 genes) had a UTR annotated. An example is provided in Fig. 2a, where the gene model in the current version of the genome (v1.0) is compared with the gene model after incorporating our analyses (indicated as v1.1, with the most notable changes indicated with orange arrows, Fig. 2). Our analyses also permitted us to improve the annotation of 3,096 gene models (Supplementary Table S4), as illustrated by *Ta1.0_00562* (Fig. 2b), in which the integration of the expression data indicates the presence of missing coding regions, in this case, represented by 3 additional exons in the middle of the CDS. Mergers between neighboring genes that were originally annotated as separate genes resulted in 196 fused gene models. One such example is provided by the gene models annotated as *Ta1.0_00791* and *Ta1.0_00792* in v1.0, which in fact corresponded to a single gene, as evidenced by the Nanopore sequencing results (Fig. 2c), and comparison with *Arabidopsis*. In addition, we identified 46 new genes (Supplementary Table S5) with robust expression support that were not represented in v1.0 of the genome, with 2 examples represented in Supplementary Fig. S3. Finally, we use the TranscriptomeAnalyzer of the NGSEP module to evaluate our annotation. We were able to identify 1,627 start/stop codons which means an improvement in 55% of the genes that did not have this information in v1.0.

**Table 2.** Comparative summary between the 2 versions of the annotation of pennycress and *Arabidopsis thaliana.*

	PC_v.1.0	PC_v1.1	*Arabidopsis*
Total genes	27,390	27,213	27,416
Average gene length	2,195.26	2,454.17	2,206.02
Average exon number	5.54	5.63	5.86
Average transcripts per gene	1	1.2	1.29
Average CDS length	1,238.99	1,254.74	1,230.62
Genes with both 5′ and 3′ UTR	1,466	10,426	27,416

As an additional control of the predicted genes, we evaluated mRNA accumulation levels by estimating transcripts per million (TPM) for each gene in each dataset. We determined that 15,700 genes had TPMs of more than 0.5 across all data sets (publicly available and embryo-derived), while 1,588 genes showed expression support only by the Illumina data derived from the 10 and 16 DAP embryos. Nanopore sequencing data provided support for additional 32 genes, which were predicted previously (v1.0), but for which there was no reported RNA-seq information. In contrast, 980 genes annotated in v1.0 were not represented in the new embryo gene expression data, and hence could not be verified (Supplementary Table S6).

In the previous annotation (v1.0), no isoforms resulting from alternative splicing were reported. In our analyses, through the splice graph implemented in the algorithm in StringTie software (Pertea *et al.*, 2015), we identified new alternative splicing isoforms for 2,842 genes, out of which 2,561 were also supported by the Nanopore data. From these, 50.2% have 2 isoforms, 29.9% have 3 isoforms, 8.5% have 4, 6.3% have 5, 4.3% between 6 and 10, and just 0.3% have >10 isoforms. We estimated the average transcript number per gene to be 1.2, similar to what was found for *Arabidopsis* and *Eutrema* (Dorn *et al.*, 2015).

To functionally annotate the pennycress genes, we used the Trinotate pipeline (Supplementary Fig. S5), in which all the CDS sequences were first evaluated with Transdecoder along with the 6 potential ORFs, 3 reading frames for each DNA strand. The predicted coding regions were then aligned with available sequences in the Uniprot (Bateman *et al.*, 2021) and Pfam (Mistry *et al.*, 2021) databases. Using this analysis, we identified 23,538 ORFs containing known protein domains present in the Pfam database. We subjected these 23,538 ORFs to GO evaluations (see *Materials and Methods*), resulting in a total of 43,640 GO terms; of those, 12,857 belonged to the biological process category, 17,995 to cellular component, and 12,788 to molecular function (Supplementary Table S7). Furthermore, we compared our GOs results with 59,023 GO annotations from *Arabidopsis*, and we found a similar number of genes in GOs categories between both organisms (Supplementary Fig. S6). When considered together, our results significantly contribute to improving the functional annotation of the pennycress genome.

### Genome diversity between the accessions

**Fig. 3.**
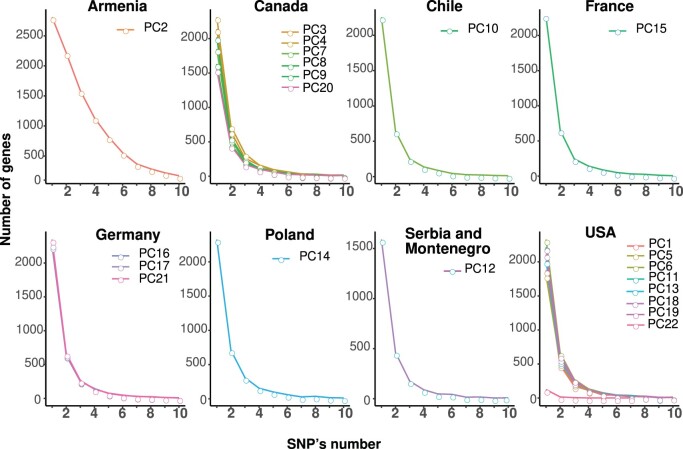
Analysis of SNP distributions among 22 pennycress accessions. In the representation, accessions were grouped according to geographical origin.

We evaluated 22 pennycress accessions of different bio-geographical origins (Table 1) for SNP discovery. From the RNA-seq data, we obtained an average of 35 million reads per accession, with about 98% of the reads mapping to the available MN106 v1.0 reference genome. The unique reads in each accession were used to variant calling with NGSEP v.4.0.0 (Tello *et al.*, 2019) identifying a total of 60,607 SNPs, out of which PC2 contributes 34,755 unique SNPs. Following the filtration step (see *Materials and Methods*), pennycress accessions were identified with on average 6,188 biallelic SNPs, with the exception of PC22 that showed only 259 SNPs, consistent with being used as the reference genome (MN106). The evaluation of SNP densities per gene showed very similar patterns across the various accessions investigated, the exception being accession PC2, which shows a much larger variation to the reference genome when compared with the others (Fig. 3a).

We determined a transition/transversion rate (Ts:Tv) of 1.5, consistent with transitions being more frequent than transversions, as has been shown in other plants. The rate, however, varies significantly across species depending on genome composition (Zhao *et al.*, 2006) and possibly growth conditions (Belfield *et al.*, 2021). For example, a similar trend was reported for *Arabidopsis* with a Ts:Tv value of 1.28 and slightly higher than maize with Ts:Tv = 1.48 (Morton *et al.*, 2009). This contrasts with the high Ts:Tv value determined for *Camelina sativa*, which was estimated at 2.80 (Luo *et al.*, 2019).

Previously, a set of 9,157 SNPs was reported (Frels *et al.*, 2019). When we analyzed these SNPs distributed across the genome, we determined that 416 were associated with annotated expressed regions, while the rest were intergenic. We compared our matrix of 60,607 SNPs with the available 416 SNPs in expressed regions (Frels *et al.*, 2019) and we found all the 416 SNPs present among the SNPs that we identified, highlighting the robustness of our approach. The number of homozygous and heterozygous SNPs was evaluated across all the accessions (Supplementary Tables S9 and S10). Based on the numbers obtained, it is evident that accessions PC3, PC4, and PC9 have higher heterozygosity than the rest of the population. It could be that these accessions have been through fewer rounds of inbreeding and hence would be less stable, compared to the rest of the population. On the other hand, 7 out of the 8 accessions from the United States showed only ∼300 polymorphic sites (Supplementary Tables S9 and S10).

**Fig. 4.**
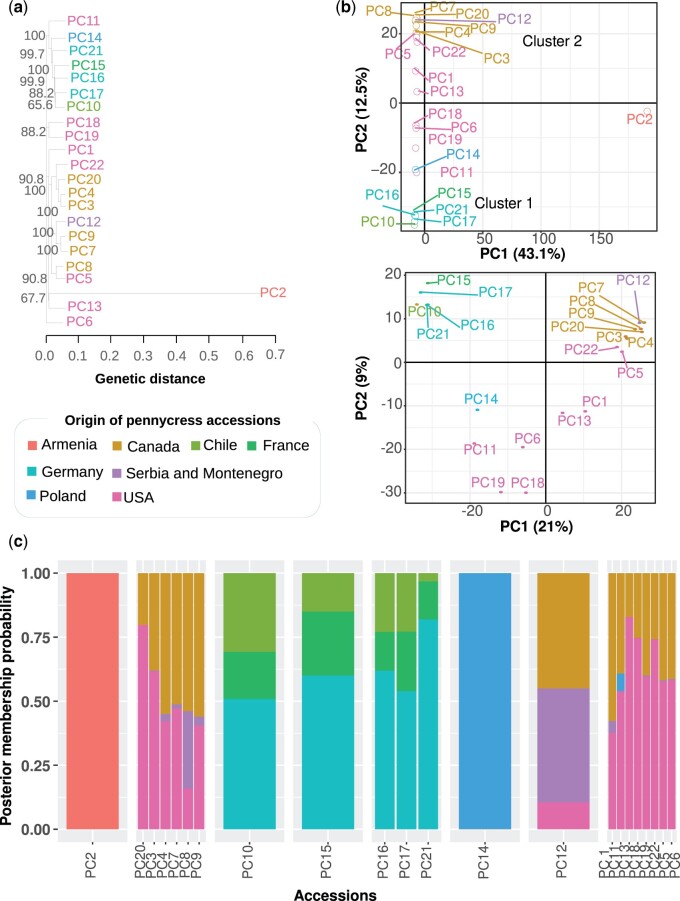
(a) Relationship between the accessions based on the presence of transcribed SNPs. The tree was constructed using the neighbor-joining (NJ) method and branch support was provided by performing 1,000 bootstrap replicates. (b) Top panel: PCA of the pennycress accessions, where the accession coming from Armenia PC2 shows a high divergence in comparison with other pennycress accessions. Bottom panel: PCA without PC2 accession coming from Armenia. (c) Clusters to study pennycress population structure through the DAPC and evaluation of the posterior membership probability for each sample to each of the predetermined populations.

We used the identified SNPs to investigate the genetic structure of the pennycress accessions studied here. Through phylogenetic tree reconstructions and principal component analysis (Fig. 4, a and b), we found that PC2 (Ames 32872) is sister to all the other accessions. In addition, the unique accession of Serbia and Montenegro (PC12) placed together with the samples coming from Canada and the United States, with the exception of PC11 (Ames 30985 belongs to South Dakota, USA) that showed more similarity to accession PC14 (Ames 22461, Poland). The remaining accessions, mainly from Europe, grouped with PC10 (Ames 30933, Magallanes-Chile). These results are consistent with previous studies (Frels *et al.*, 2019), and support the use of RNA SNPs as good markers for population diversity, without the need to perform whole-genome sequencing (Takahagi *et al.*, 2016; Rogier *et al.*, 2018). STRUCTURE analyses showed at *K* = 2 the division between non-Armenian and Armenian accessions while the remaining evaluated *K* values failed to show additional new groups (Supplementary Fig. S7).

To further investigate the structure of the dataset, we removed the Armenian sample and re-evaluated the data with STRUCTURE (Supplementary Fig. S8). At *K* = 2, the first cluster harbors the samples from PC10 (Chile), PC15 (France), PC16, PC17, and PC21 (Germany), and the remaining of the other cluster. At *K* = 3, the accessions PC10, PC15, PC16, PC17, and PC21 remained in a cluster, while accessions PC7, PC8, PC9, and PC20 (from Canada) presented to probability close to 80% belonging to a new cluster. In addition to PC14, PC13, PC18, and PC19 originating from the United States maintained a 100% probability of belonging to the same cluster. At *K* = 4, PC4, PC7, PC8, PC9, PC12, and PC13 show admixed between populations. However, the accessions from Chile, France, and Germany show less than 70% membership in this population when evaluated at *K* = 5. We conclude from this analysis that by removing PC2, new group relationships appear according to the geographic origin of the accessions. However, some accessions such as PC10 and PC16 show an admixed pattern regardless of the *K* value used.

Discriminant analysis of principal components through Bayesian clustering methods allowed us to evaluate a probabilistic assignment of individuals to each pre-defined group according to accession origin (Fig. 4c). The PC2 and PC14 accessions show a posterior membership probability of 1, indicating a high likelihood of belonging to the reported origin, Armenia and Poland, respectively. Samples coming from Canada (gold color, Fig. 4c) exhibit an admixture pattern between the United States (pink color) and the sample from Serbia and Montenegro (purple color), except for PC20 that presented a posterior probability of ∼0.79 to belong to the United States and 0.21 to Canada. Similar trends show that the PC13, PC18, and PC22 samples were derived most likely from the United States. From this same group, PC11 showed a small probability (0.08) of belonging to Poland. The remaining samples originating in the United States showed probabilities of less than 0.5 for belonging to Canada. Finally, we found an interesting admixture pattern with PC10 (Chile), PC15 (France), and PC16, PC17, and PC21 (Germany). These patterns can be explained according to the pennycress origin in Eurasia (Best and McIntyre 1975, Hume 1990, Warwick *et al.*, 2002), and their subsequent migration from Europe to the American continent, starting in Canada where it was collected around 1860 and later distributed throughout the United States (Mitich, 1996). The trends identified for pennycress are similar to what has been determined for *Arabidopsis*, for which a positive correlation between genetic variation and the geographic origin of populations has been determined (Shindo *et al.*, 2005).

### Evaluation of likely signatures of natural selection

SNPs are important molecular markers to analyze natural genetic variation within species, and they are widely used to identify potential correlations between genes, phenotypic variation, and adaptation to various environments (Shindo *et al.*, 2005; Mitchell-Olds and Schmitt, 2006). To detect possible signatures of natural selection, we applied the fixation index (*Fst*), which compares the variance of allele frequencies among populations. A high *Fst* value indicates that allele frequencies are different between 2 populations, while a small *Fst* value means that the allele frequencies within each population are similar (Holsinger and Weir, 2009; Vitti *et al.*,2013). We used the Tajima’s *D* test to summarize the site frequency spectrum by assessing the pairwise differences with the number of segregating sites in each gene (Ferretti *et al.*, 2018). A *D* value of 0 indicates neutrality, a negative value suggests an excess of rare variations, selective sweeps, or positive selection, and a positive value indicates balancing selection with intermediate allele frequencies (Carlson *et al.*, 2005). Both tests were performed to identify likely candidate genes that have been the target of selection.

As the Armenian accession PC2 presented such a distinctive set of SNPs (Fig. 4), this line was not included in any of the subsequent analyses. According to the PCA without PC2, 2 separate populations were identified: cluster 1 containing lines from Canada, the United States, Poland, and former Serbia and Montenegro (PC1, PC3, PC4, PC5, PC6, PC7, PC8, PC9 PC11, PC12, PC13, PC14, PC18, PC19, PC20, and PC22) and cluster 2 containing lines from Germany, France, and Chile (PC10, PC15, PC16, PC17, and PC21). For these 2 groups, we estimated the *Fst* and Tajima’s *D* tests. Considering that the maximum differentiation corresponds to an *Fst* value of 1 and each population has fixed alternate alleles, only genes that presented *Fst* ≧ 0.8 were evaluated. We identified 246 genes that showed different positive selective pressure between cluster 1 (enriched in Canadian and American lines) and the main European accessions in cluster 2 (Supplementary Table S11). We applied the Tajima’s *D* test to assess pairwise differences with the number of segregating sites in each gene. Of the 246 genes, a negative Tajima’s *D* value was found in 50 genes belonging to lines associated with cluster 1, and in 217 genes belonging to lines associated with cluster 2. We found signatures of positive selection in 32 genes that belonged to both clusters 1 and 2, with 4 genes (v1.0: *Ta1.0_00784*; v1.1: *Ta1.1_00241*, v1.0: *Ta1.0_00708*; v1.1: *Ta1.1_00165*, v1.0: *Ta1.0_03845*; v1.1: *Ta1.1_04900*, v1.0: *Ta1.0_05649*; v1.1: *Ta1.1_04678*) showing values below −2 (Supplementary Fig. S9).

We then characterized through functional enrichment analysis the 246 genes to infer potential biological functions (Supplementary Table S12). The biological categories of cellular processes (GO: 0009987) and primary metabolic processes (GO: 0044238) were the most significantly enriched (False Discovery Rate, FDR < 2 × 10^−5^). Specifically, we identified genes related to the metabolism of amino acids such as asparagine synthetase (v1.0: Ta1.0_02601; v1.1: *Ta1.1_03269*), glutamate synthase (v1.0: *Ta1.0_21588*; v1.1: *Ta1.1_19946*), acetylornithine aminotransferase (v1.0: *Ta1.0_08963*; v1.1: *Ta1.1_06945*) and arginine decarboxylase (v1.0: Ta1.0_11720; v1.1: *Ta1.1_08329*); polysaccharide metabolism such as phosphoglucomutase (v1.0: *Ta1.0_17386*; v1.1: *Ta1.1_20514*), starch branching enzyme (v1.0: *Ta1.0_16017*; v1.1: *Ta1.1_14558*), and UDP-xylose synthase (v1.0: T*a1.0_10496*; v1.1: *Ta1.1_09740*); and lipid biosynthesis such as the plastid E1b pyruvate dehydrogenase subunit (v1.0: *Ta1.0_06432*; v1.1: *Ta1.1_07086*) and homomeric acetyl-CoA carboxylase (v1.0: *Ta1.0_22926*; v1.1: *Ta1.1_24033*). These results suggest that central metabolism may have evolved in response to adapting to distinct environments.

## Conclusion

Our studies provide a significant improvement of the pennycress genome annotation by combining short- and long-reads in the context of the available genome sequence information. We identified a large number of transcribed SNPs, providing on average over 6,000 bi-allelic SNPs to differentiate between the 22 accessions analyzed. Our results clearly suggest that the Armenian accession PC2 differentiates itself significantly from the others. Furthermore, through the SNPs identified, we evaluated the population structure of our accessions and assessed the data for signatures of natural selection. We identified an interesting set of candidate protein-coding genes under likely positive selection in pennycress, and functional enrichment analysis implicated 7 of those genes with the metabolism of amino acids and lipids. These allelic variants under selection pressure provide potential targets for research toward pennycress genome engineering and optimization. We considered that using transcribed SNPs for the evaluation of signatures of natural selection provided an attractive approach to understand the possible evolutionary forces that could have shaped pennycress populations. However, it is crucial in future research to consider whole-genome resequencing of pennycress accessions to explore other genomic segments, particularly regulatory regions that have previously been shown to be important for crop domestication and improvement (Meyer and Purugganan, 2013; Swinnen *et al.*, 2016).

## Data availability

The data underlying this article are available with the BioProject ID PRJNA751040. Besides, the scripts used are available on GitHub: https://github.com/Thlaspiarvense/Pennycress.

Supplemental material is available at *GENETICS* online.

## Supplementary Material

jkac084_Figure_S1

jkac084_Figure_S2

jkac084_Figure_S3

jkac084_Figure_S4

jkac084_Figure_S5

jkac084_Figure_S6

jkac084_Figure_S7

jkac084_Figure_S8

jkac084_Figure_S9

jkac084_Supplemental_Material

jkac084_Table_S1

jkac084_Table_S2

jkac084_Table_S3

jkac084_Table_S4

jkac084_Table_S5

jkac084_Table_S6

jkac084_Table_S7

jkac084_Table_S8

jkac084_Table_S9

jkac084_Table_S10

jkac084_Table_S11

jkac084_Table_S12
