# Supplementary figures and images for: Natural variation and improved genome annotation of the emerging biofuel crop field pennycress (Thlaspi arvense)
Source: G3 (Bethesda). 2022 Apr 13;12(6):jkac084. doi: 10.1093/g3journal/jkac084 (PMC9157065; doi:10.1093/g3journal/jkac084)

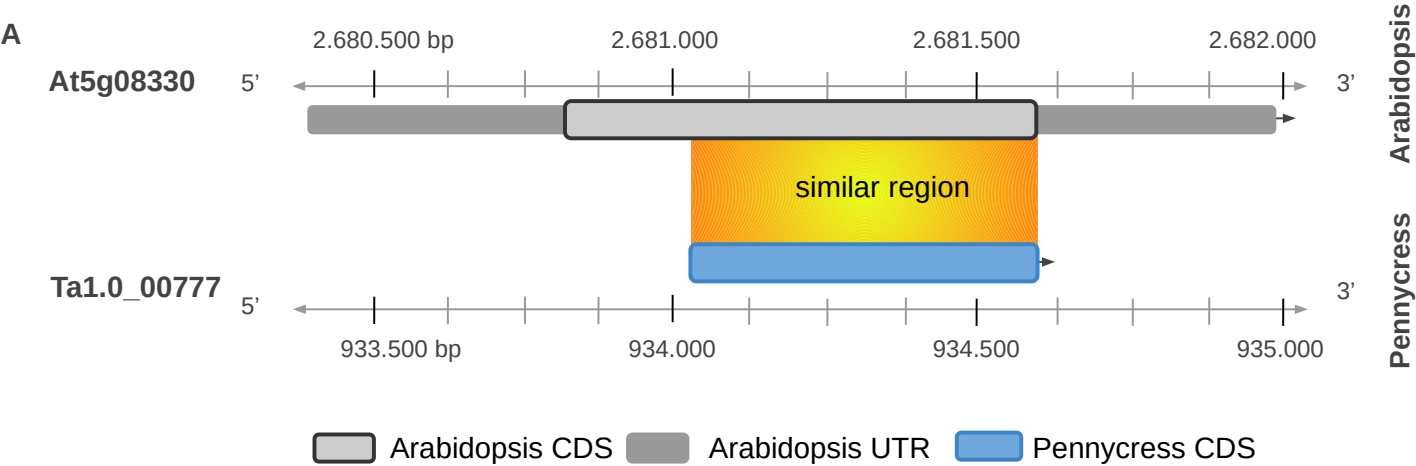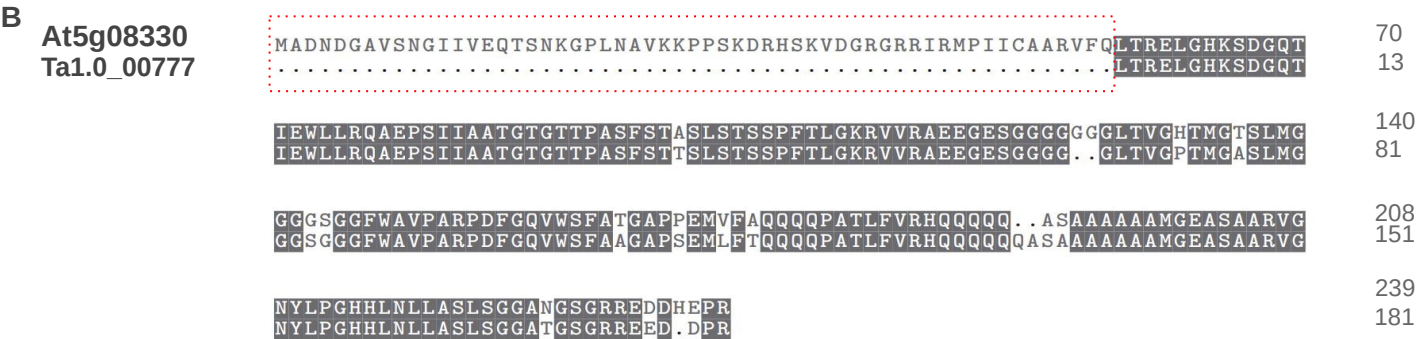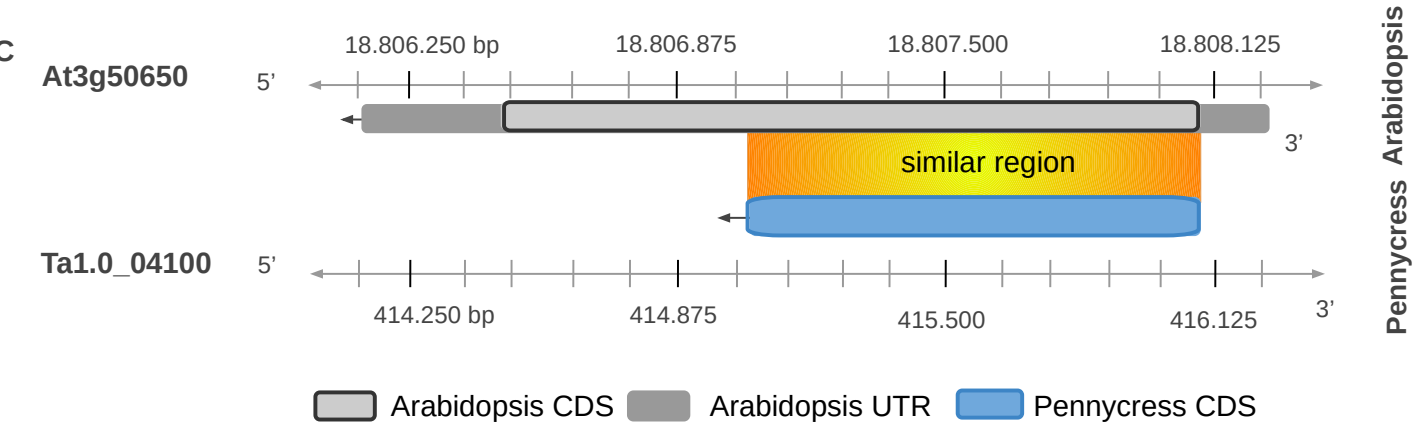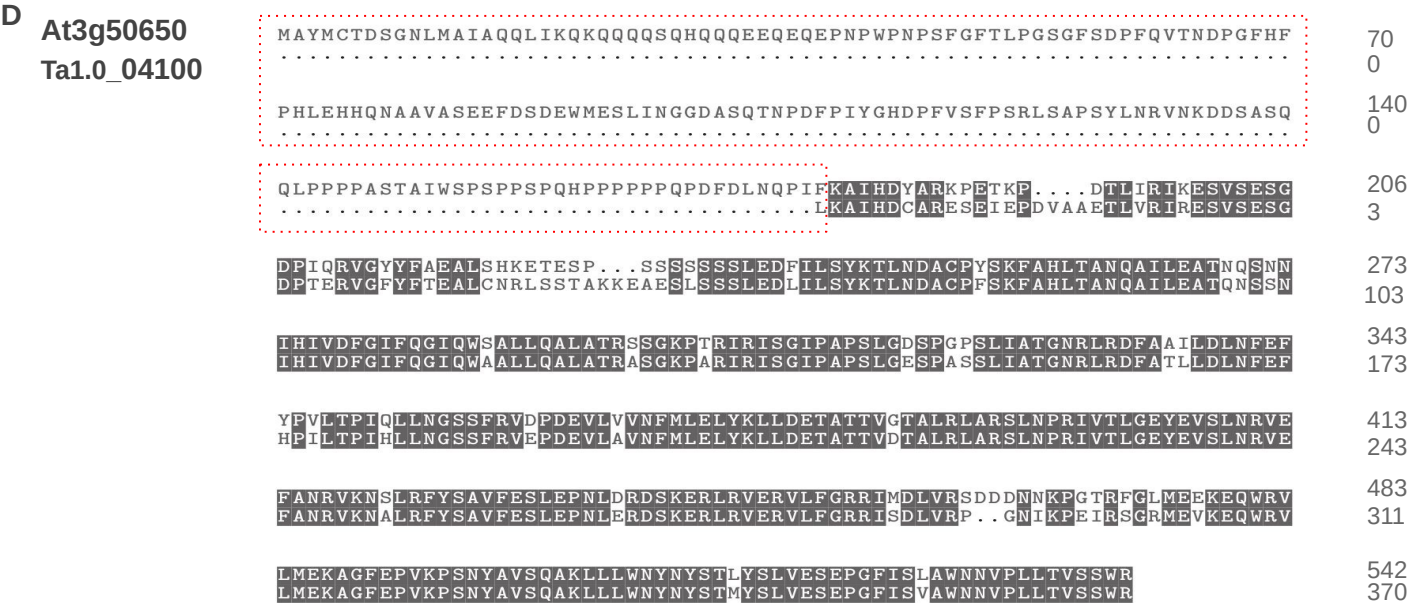

Supplement: jkac084_Figure_S1 [file jkac084_figure_s1.pdf]

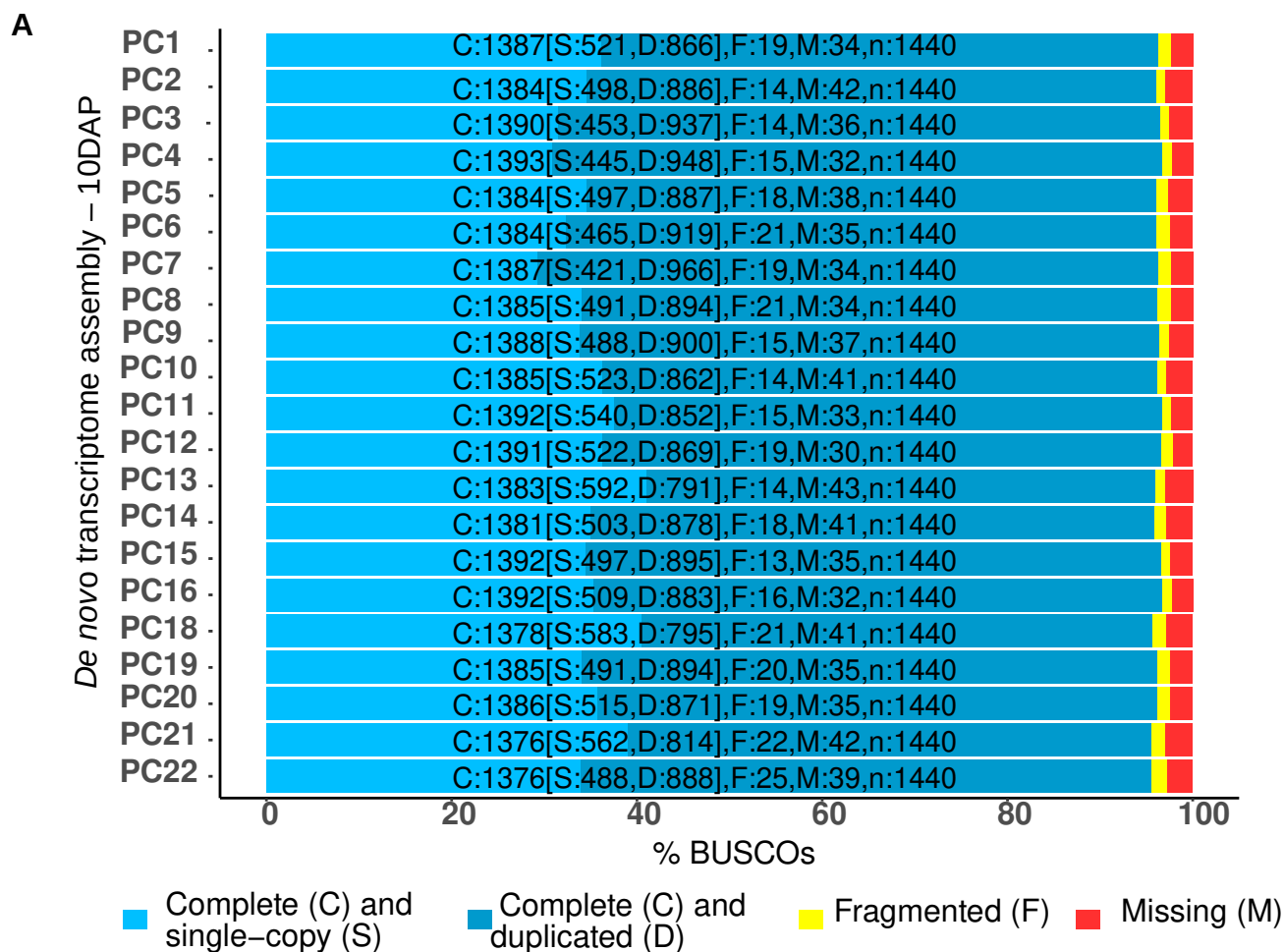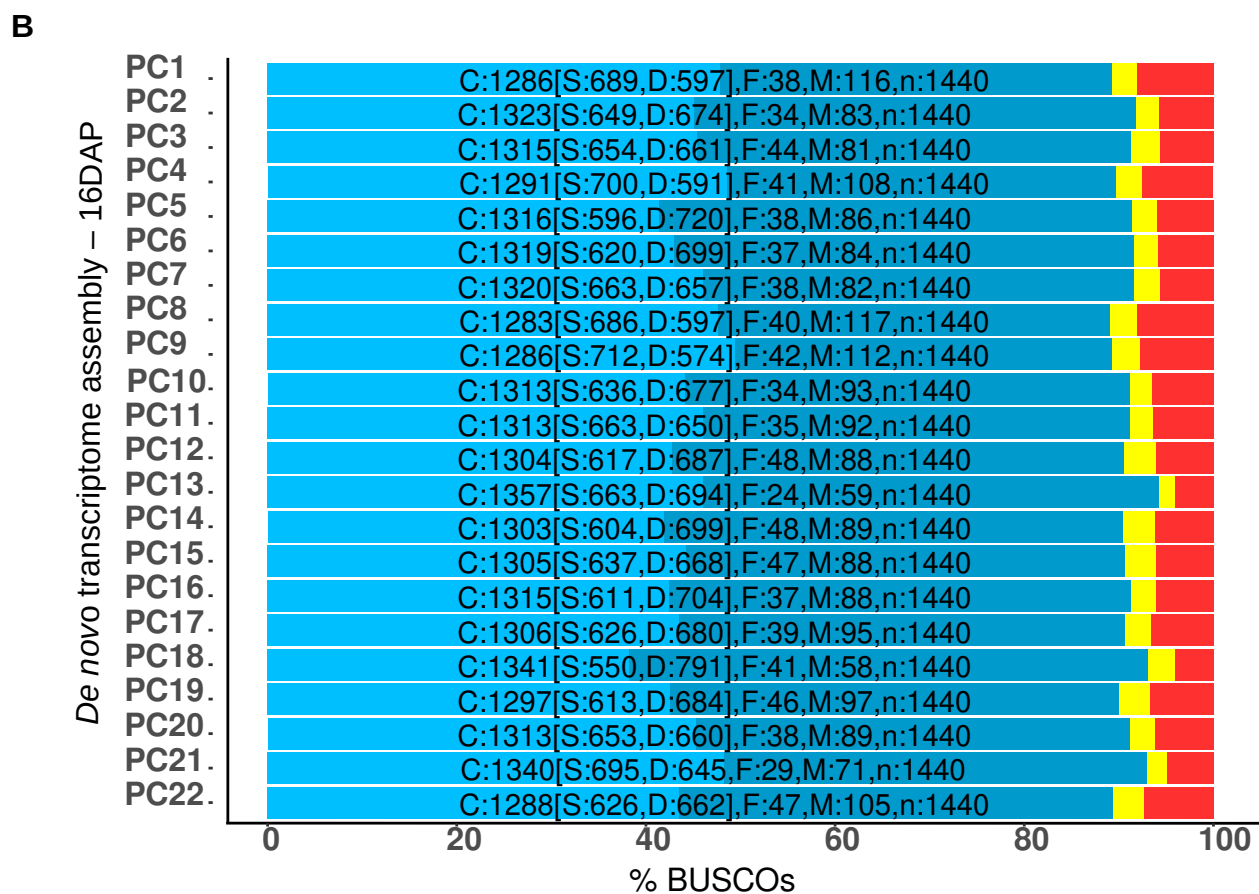

Supplement: jkac084_Figure_S2 [file jkac084_figure_s2.pdf]

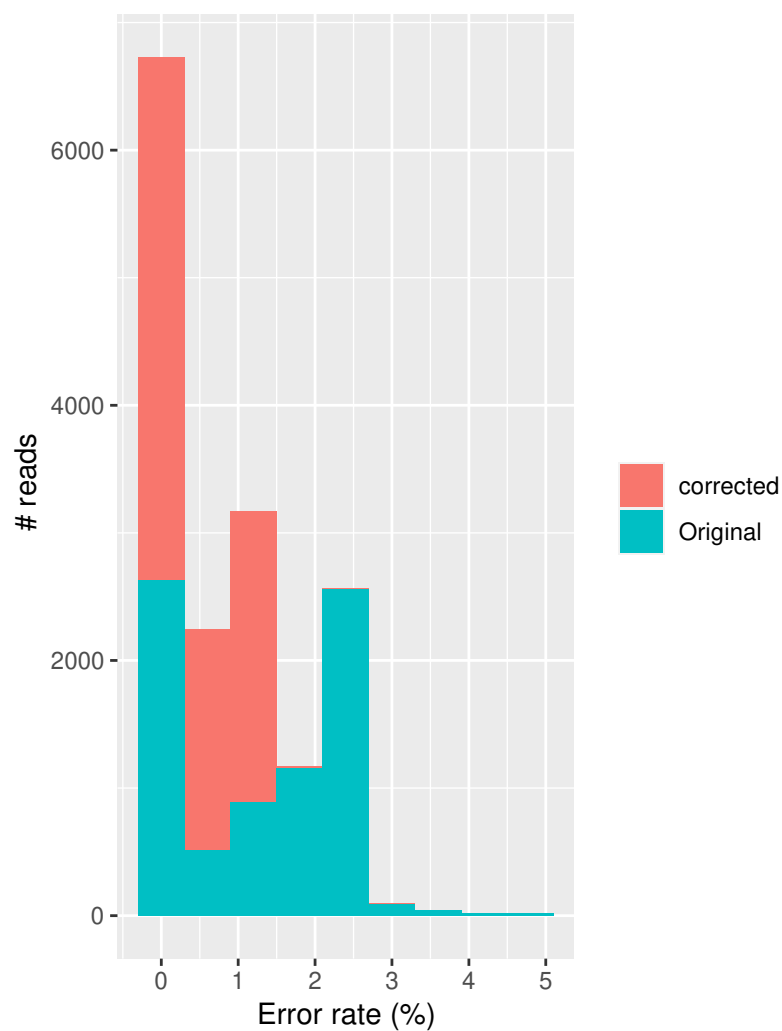

Supplement: jkac084_Figure_S3 [file jkac084_figure_s3.pdf]

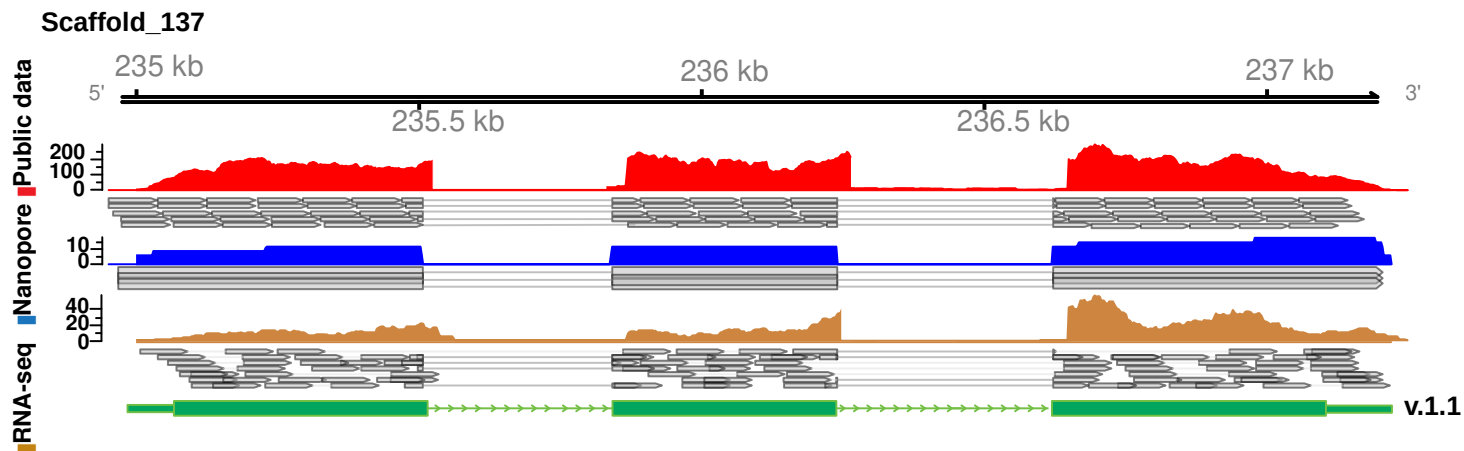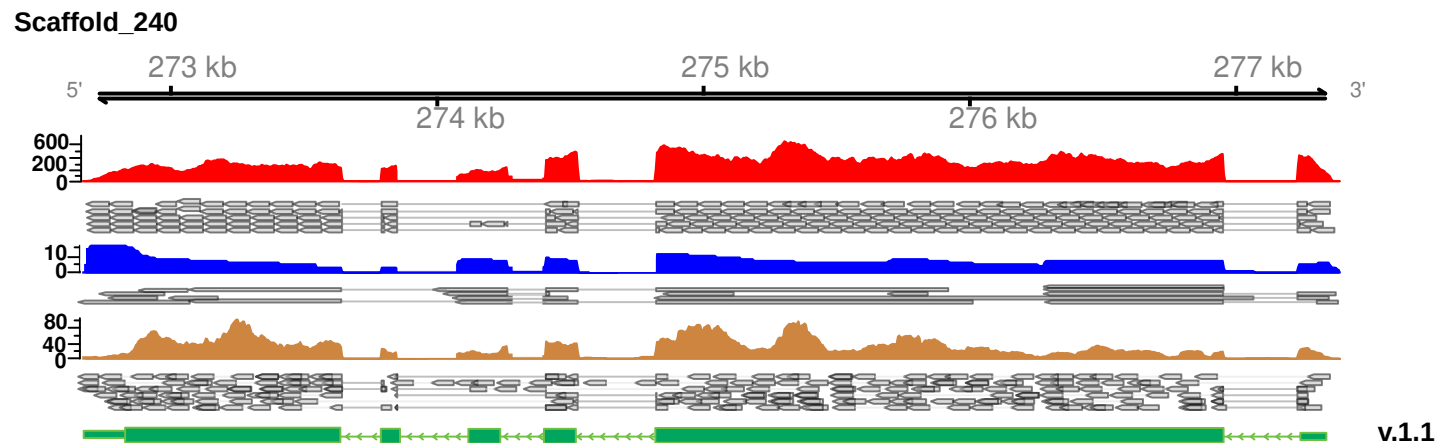

Supplement: jkac084_Figure_S4 [file jkac084_figure_s4.pdf]

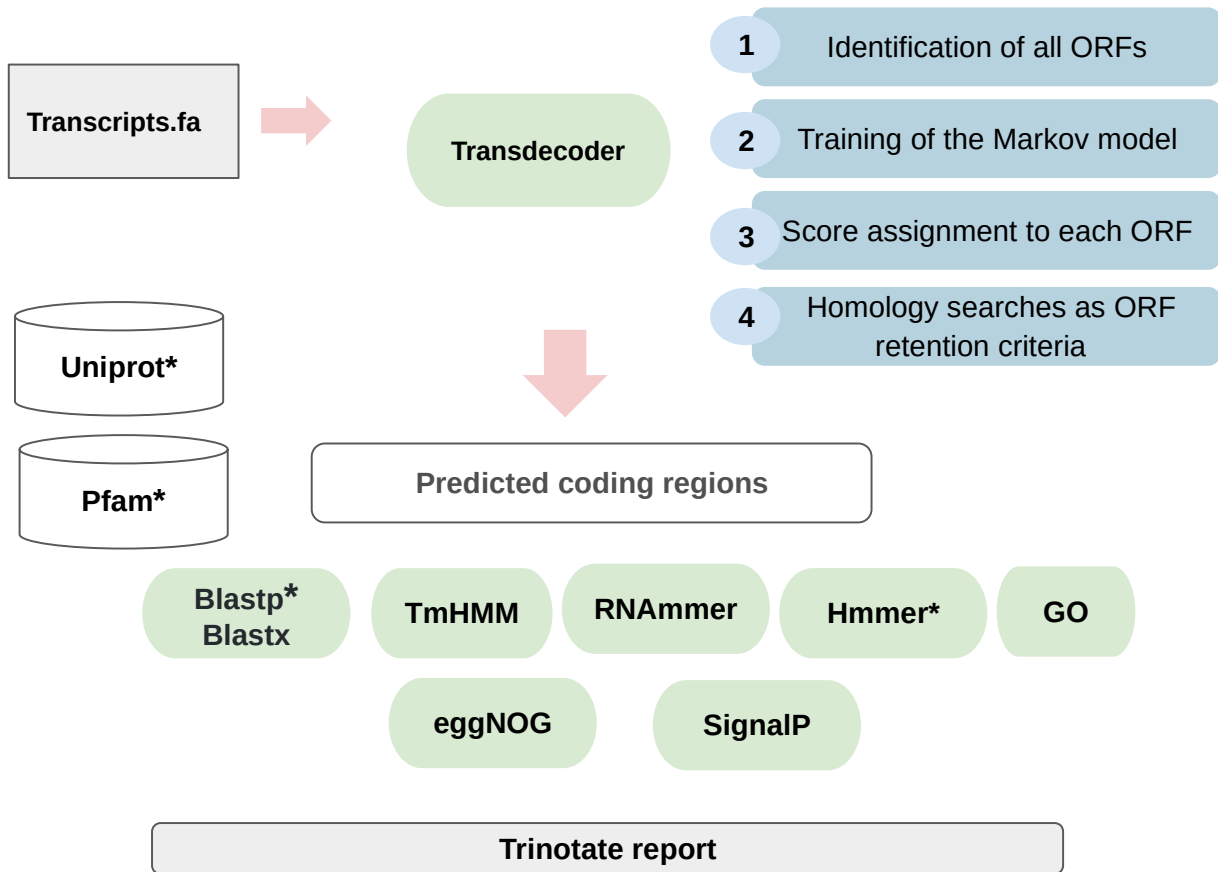

Supplement: jkac084_Figure_S5 [file jkac084_figure_s5.pdf]

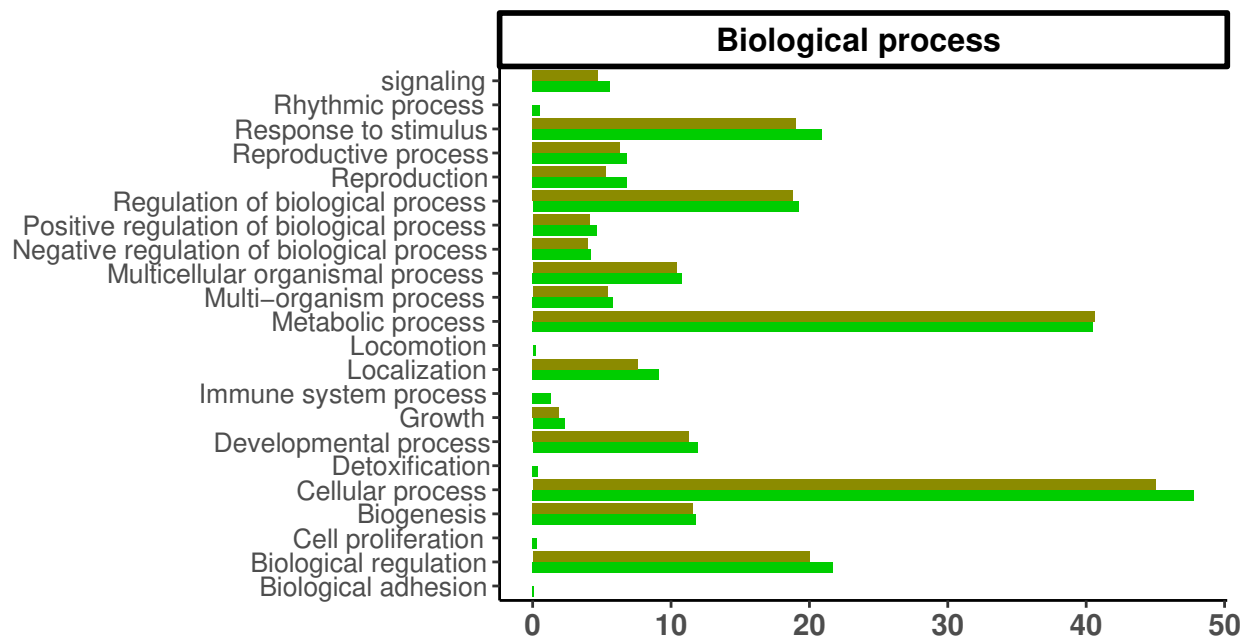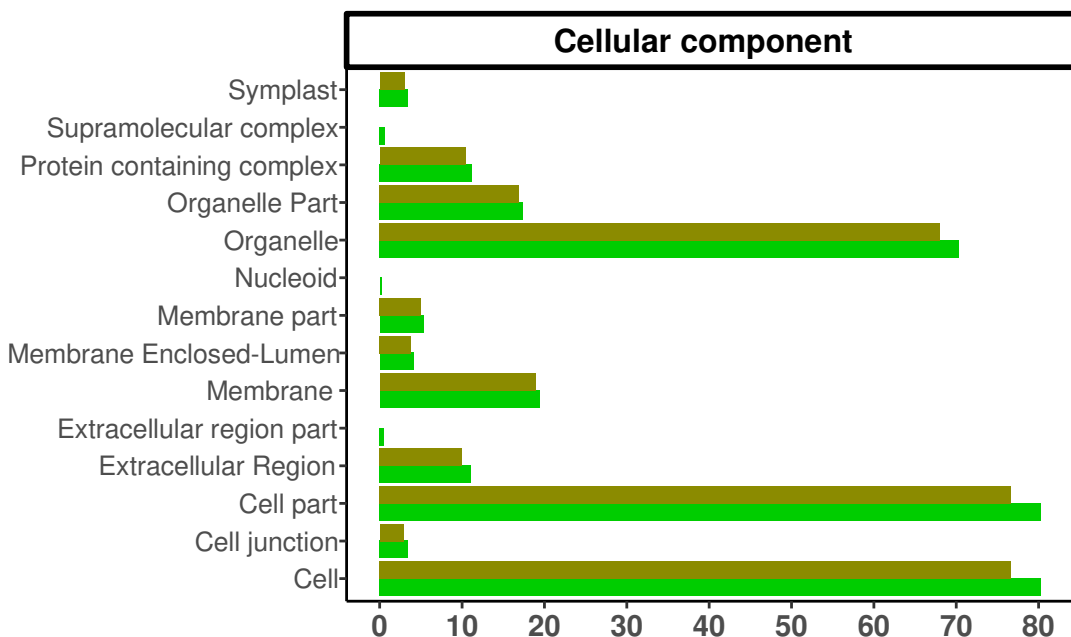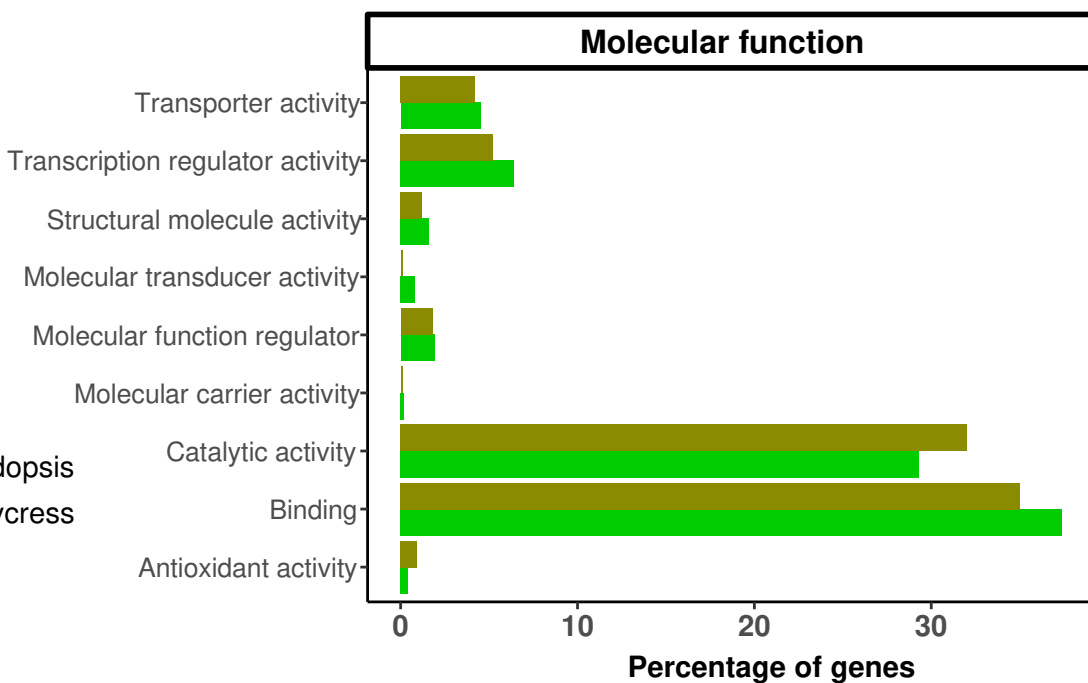

Specie

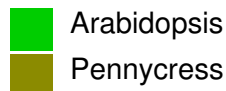

Percentage of genes

Supplement: jkac084_Figure_S6 [file jkac084_figure_s6.pdf]

**K=2**

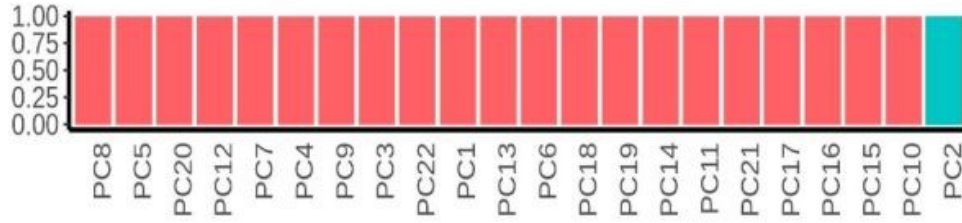

**K=3**

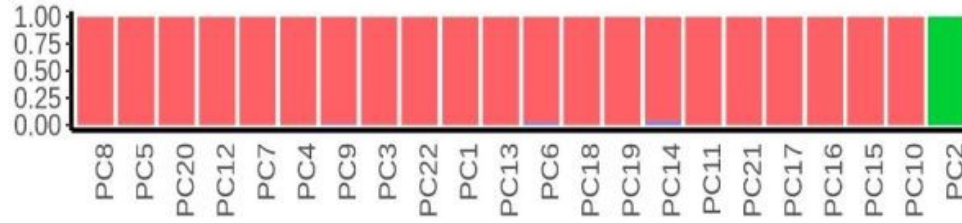

**K=4**

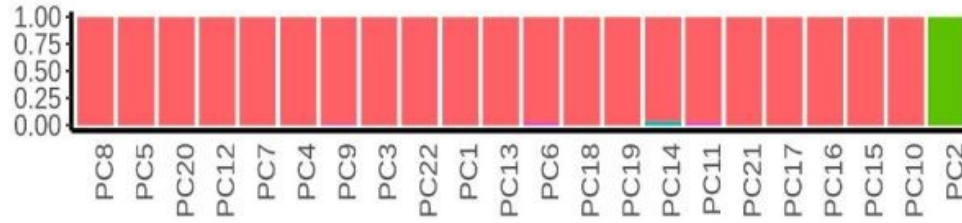

**K=5**

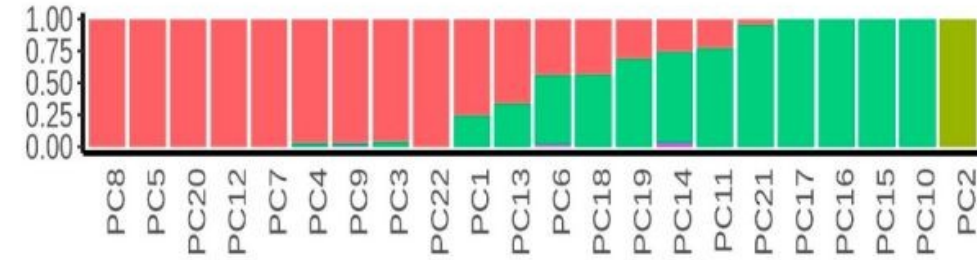

Supplement: jkac084_Figure_S7 [file jkac084_figure_s7.pdf]

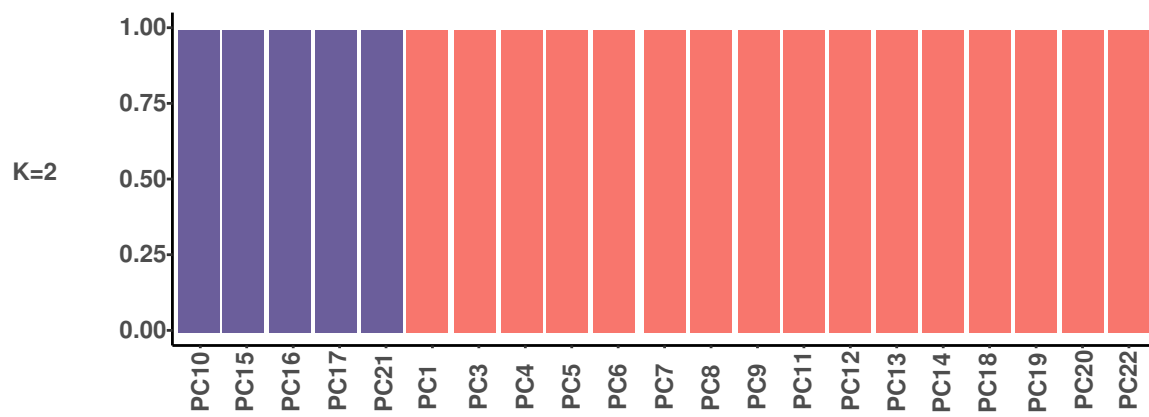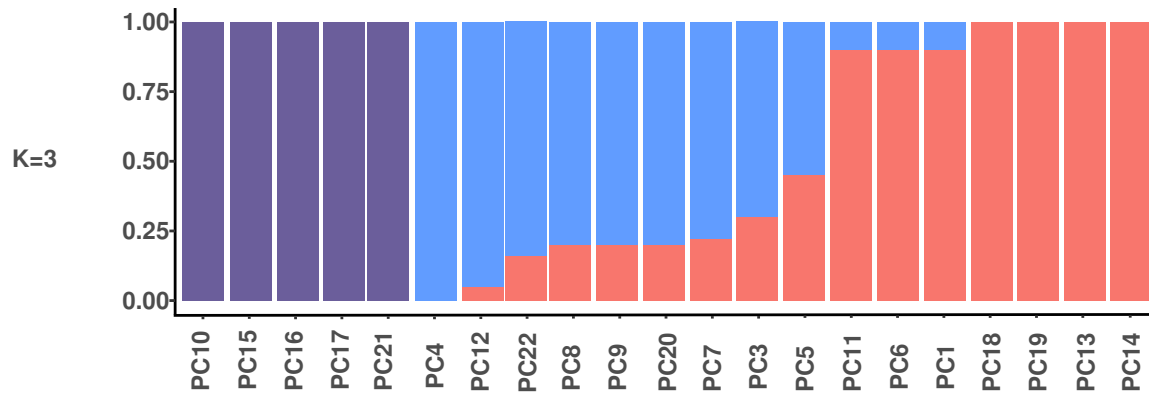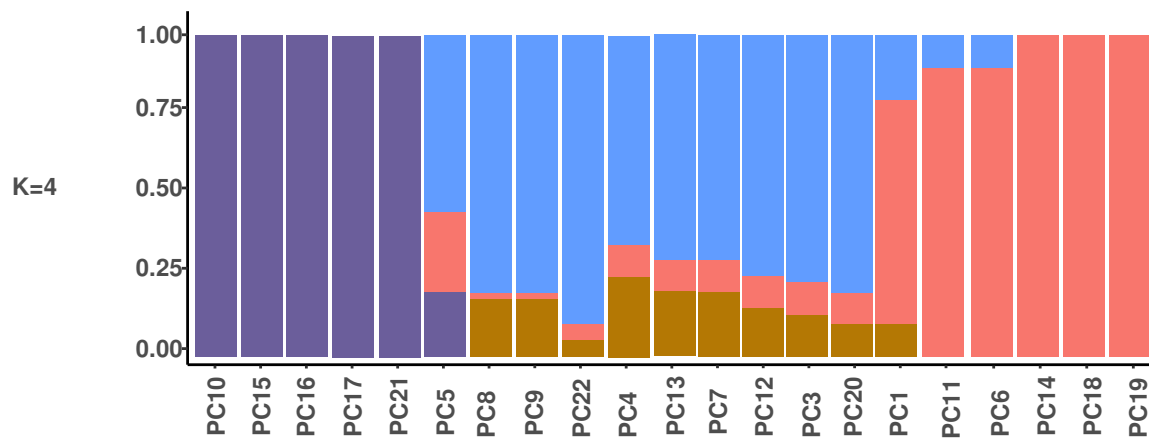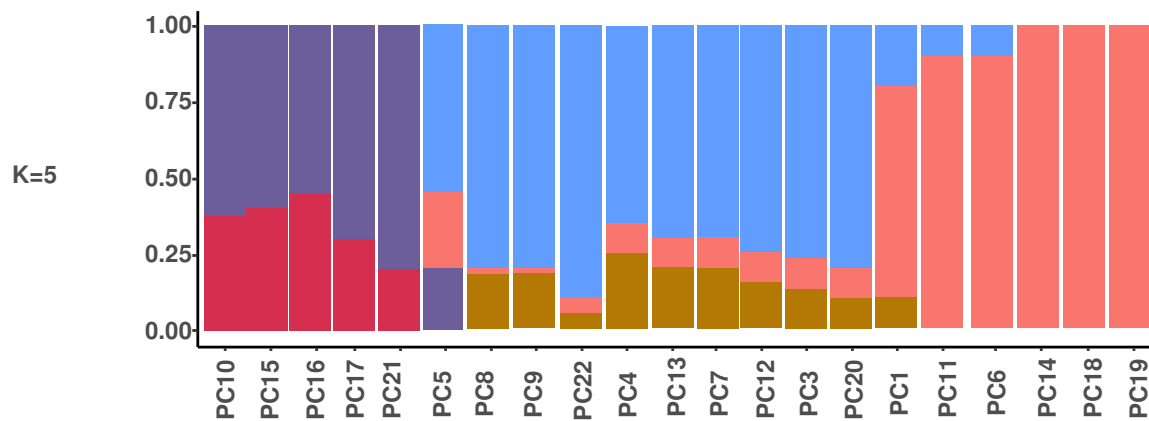

Supplement: jkac084_Figure_S8 [file jkac084_figure_s8.pdf]

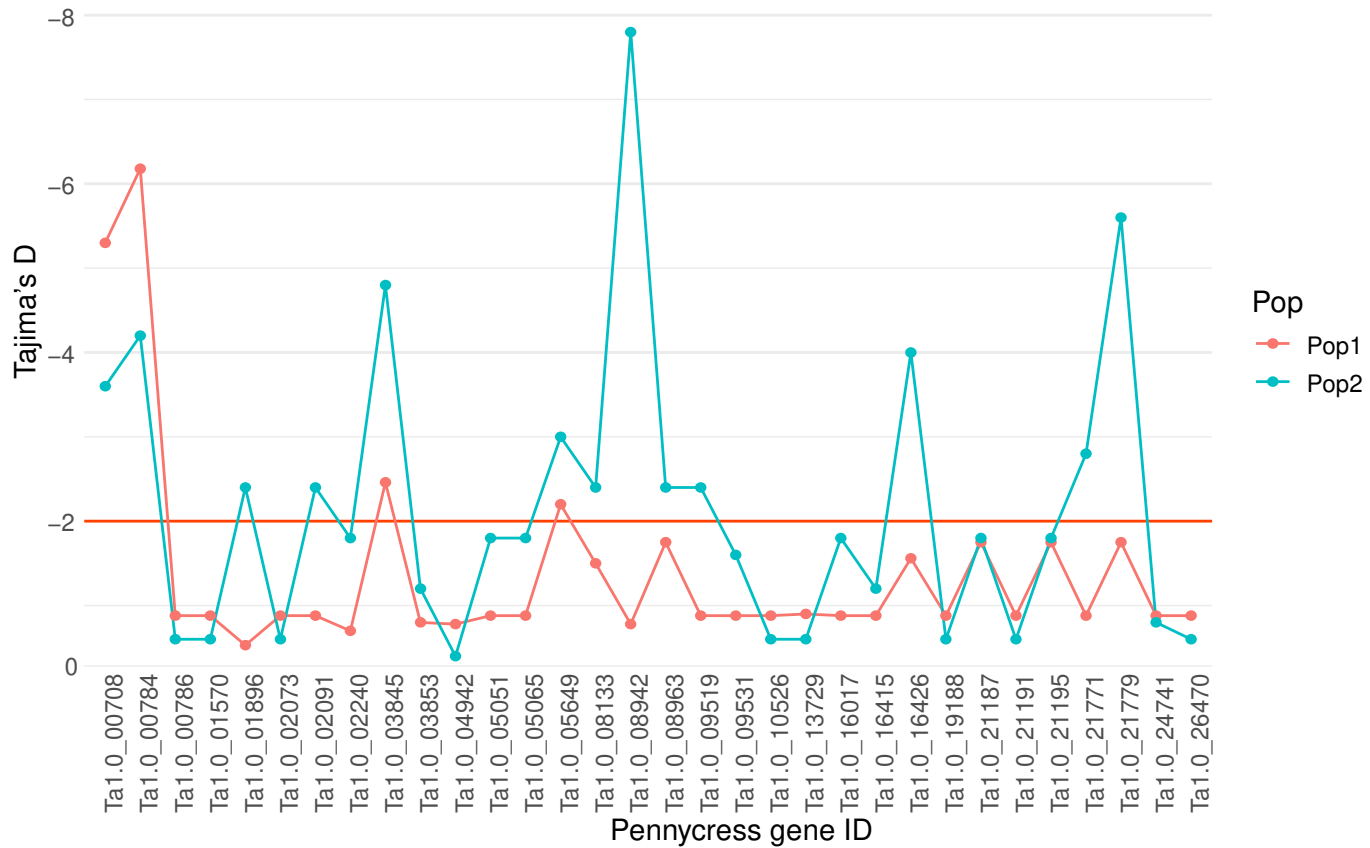

Supplement: jkac084_Figure_S9 [file jkac084_figure_s9.pdf]
