# Supplementary material for: Natural variation and improved genome annotation of the emerging biofuel crop field pennycress (Thlaspi arvense)
Source: G3 (Bethesda). 2022 Apr 13;12(6):jkac084. doi: 10.1093/g3journal/jkac084 (PMC9157065; doi:10.1093/g3journal/jkac084)
Supplement: jkac084_Supplemental_Material [file jkac084_supplemental_material.docx]

**Supplementary figures.**

**Figure S1.** Examples of pennycress genes lacking canonical ATG start codons in v1.0 of the genome. The similar region in orange color, represent the part of aligment where was detecting similarities between sequences by paired alignment of amino acid sequences between pennycress and Arabidopsis genes. The red dotted box shows the initial part of the pennycress gene missing. A) and C) Gene models in *Arabidopsis thaliana* and pennycress. B) Amino acid alignment between pennycress Ta1.0_00777 with the orthologous gene in *Arabidopsis thaliana* At5g08330. D) Amino acid alignment between Ta1.0_04100 and At3g50650.

**Figure S2.** BUSCO summary results for the *de novo* transcriptome assemblies. The letter C shows the total number of complete genes, which could be single-copy (S) or duplicated (D), represented in light blue (S) and dark blue (D) colors respectively. Fragmented genes (F) are indicated in yellow color, and Missing (M) genes are indicated in red. (n) The total genes in Embryophyta library OrthoDB v8 used in the analysis is indicated by n.

**Figure S3.**  Evaluation of the error rate of the original and corrected nanopore reads. In blue color are the original reads and in pink the corrected reads.

**Figure S4.** Two new genes were identified from the gene expression data. The gene model is represented by the green boxes. The red track indicates the read distribution corresponding to previously available RNA-seq data, reads obtained from Nanopore are shown in blue, and the embryo reads from the Illumina sequencing platform in golden. In v.1.0 of the pennycress genome, those genes were not present.

**Figure S5.** Functional annotation workflow. We used Transdecoder to first evaluate the transcripts and predict the best coding regions by the steps indicated by the four blue squares. Next, the predicted coding regions were used as input in several algorithms represented by the green colors. The different outputs were then used by Trinotate to generate an annotation report. The Uniprot and Pfam databases were used as "subject" in the algorithms marked with asterisks.

**Figure S6.** Gene ontology terms identified in pennycress coding genes. A comparison with *Arabidopsis* is shown and gene ontology terms were divided into biological process, cellular components, and molecular function categories,

**Figure S7.** Estimation of population structure using the STRUCTURE package involving Bayesian cluster analysis. The K letter indicates the number of populations. Each pennycress accession is represented by a vertical line. The colors represent the likelihood that each accession belongs to a particular population.

**Figure S8.** Estimation of population structure without PC2 using the STRUCTURE package involving Bayesian cluster analysis. The K letter indicates the number of populations. Each pennycress accession is represented by a vertical line. The colors represent the likelihood that each accession belongs to a particular population.

**Figure S9.** Estimation of Tajima’s D test.

**Supplementary tables.**

**Table S1.** Summary statistics of the Nanopore sequencing results.

**Table S2**. Raw counts and alignment rate by each sample.

**Table S3.** Description of the public data used in this study.

**Table S4.** Modifications of the existing pennycress gene models.

**Table S5.** IDs for pennycress new genes.

**Table S6.** Transcripts per million (TPM) per gene in each data set.

**Table S7.** Gene ontology terms of pennycress genes in v1.1.

**Table S8.** Full-length transcripts identified by long-reads.

**Table S9.**  Number of raw number of SNPs identified in each accession with respect to MN106.

**Table S10.** Number of SNPs identified in each accession with respect to MN106.

**Table S11.** Likely genes under selection.

**Table S12.** Gene enrichment analysis of likely genes under selection.
